# Influence of Oligomeric Lactic Acid and Structural Design on Biodegradation and Absorption of PLA-PHB Blends for Tissue Engineering

**DOI:** 10.3390/polym16212969

**Published:** 2024-10-23

**Authors:** Jana Čajková, Marianna Trebuňová, Marcel Modrák, Gabriela Ižaríková, Darina Bačenková, Tomáš Balint, Jozef Živčák

**Affiliations:** 1 Department of Biomedical Engineering and Measurement, Faculty of Mechanical Engineering, Technical University of Košice, 042 00 Košice, Slovakia; marianna.trebunova@tuke.sk (M.T.); marcel.modrak@tuke.sk (M.M.); darina.bacenkova@tuke.sk (D.B.); tomas.balint@tuke.sk (T.B.); jozef.zivcak@tuke.sk (J.Ž.); 2 Department of Applied Mathematics and Informatics, Faculty of Mechanical Engineering, Technical University of Košice, Letná 9, 042 00 Košice, Slovakia; gabriela.izarikova@tuke.sk

**Keywords:** PLA/PHB polymer mixture, scaffold, 3D printing, in vitro, biodegradation

## Abstract

The advancing development in biomaterials and biology has enabled the extension of 3D printing technology to the bioadditive manufacturing of degradable hard tissue substitutes. One of the key advantages of bioadditive manufacturing is that it has much smaller design limitations than conventional manufacturing and is therefore capable of producing implants with complex geometries. In this study, three distinct blends of polylactic acid (PLA) and polyhydroxybutyrate (PHB) were produced using Fused Deposition Modeling (FDM) technology. Two of these blends were plasticized with oligomeric lactic acid (OLA) at concentrations of 5 wt% and 10 wt%, while the third blend remained unplasticized. Each blend was fabricated in two structural modifications: solid and porous. The biodegradation behavior of the produced specimens was examined through an in vitro experiment using three different immersion solutions: saline solution, Hank’s balanced salt solution (HBSS), and phosphate-buffered saline (PBS). All examined samples were also subjected to chemical analysis: atomic absorption spectroscopy (AAS), scanning electron microscopy (SEM), and energy-dispersive spectrometry (EDS). The results of the degradation experiments indicated a predominantly better absorption capacity of the samples with a porous structure compared to the full structure. At the same time, the blend containing a higher concentration of OLA exhibited enhanced pH stability over the evaluation period, maintaining relatively constant pH values before experiencing a minor decline at the end of the study. This observation indicates that the increased presence of the plasticizer may provide a buffering effect, effectively mitigating the acidification associated with material degradation.

## 1. Introduction

The increasing need for sustainable and biocompatible materials in medical applications has fueled research into biodegradable polymers, particularly for hard tissue engineering. Poly(lactic acid) (PLA) and Poly(3-hydroxybutyrate) (PHB) have emerged as key candidates for the bioadditive manufacturing of degradable hard tissue substitutes due to their biodegradability, biocompatibility [1], and mechanical properties. PLA, a widely used aliphatic polyester derived from renewable resources, is favored for its processability and strength, but its inherent brittleness limits its application in load-bearing environments. PHB, on the other hand, is known for its superior biodegradability and biocompatibility, though it suffers from mechanical limitations when used independently [2,3,4]. However, a key challenge in the development of PLA-PHB-based scaffolds for hard tissue applications lies in their stiffness and brittleness, which can be problematic for replicating the mechanical properties of bone [5]. The morphology, thermal properties, mechanical performance, and biodegradation behavior of PLA/PHB blends have been extensively studied [6,7]. Results by Zhang et al. demonstrated that while PLA and PHB are immiscible, they exhibit notable molecular interactions. PHB’s high crystallinity enhances the recrystallization of PLA, leading to an increase in the heat distortion temperature. The addition of PLA to PHB improves the mechanical strength of the PHB matrix. Conversely, blending PLA with PHB has been shown to enhance the mechanical properties of PLA as well, with the PLA/PHB 75/25 blend exhibiting significantly improved tensile strength compared to pure PLA. This improvement is attributed to the finely dispersed PHB crystals acting as both filler and nucleating agents within the PLA matrix [8]. Furthermore, biodegradation studies, based on weight loss measurements at room temperature, indicated that the biodegradability of the blends improved with increasing PHB content [9]. To further address these challenges, plasticizers such as oligomeric lactic acid (OLA) are introduced to improve the flexibility and ductility of the polymer blends. OLA, being biodegradable and biocompatible, reduces brittleness without compromising the material’s degradation profile. The concentration of plasticizer, however, must be carefully optimized to balance mechanical performance with degradation kinetics, especially in the context of hard tissue regeneration, where mechanical stability is critical during the healing process [5,10]. The study by Arrieta et al. developed flexible electrospun mats based on PLA blended with 25 wt% PHB and plasticized with different concentrations of OLA. The addition of OLA improved PLA’s crystallization and reduced the fiber diameters due to lower solution viscosity [11]. Trebuňová et al. focused on how plasticizers impacted the material’s biodegradation and interaction with 7F2 osteoblast cells (*Mus musculus*), assessing cell viability, proliferation, morphology, and surface deposition. The results showed a significant influence of plasticizer type and concentration on the material’s properties. Of the mixtures tested, the blend containing 25% OLA was found to be the most biocompatible with 7F2 osteoblasts, indicating its potential for biomedical applications [5]. These results underscore the importance of carefully controlling and optimizing the amount of plasticizers, such as OLA, in PLA-PHB blends. A delicate balance must be maintained to enhance material properties without undermining mechanical integrity or biocompatibility [11].

Additive manufacturing technologies, especially Fused Deposition Modeling (FDM), have gained prominence for fabricating custom, complex scaffolds with controlled porosity, allowing for the precise design of structures that mimic the mechanical and biological characteristics of natural bone. These scaffolds not only provide mechanical support but also facilitate cell attachment and proliferation, critical for successful tissue regeneration [12,13,14].

In tissue engineering, especially for hard tissues, biodegradability is a critical factor [15]. Scaffolds must degrade at a rate that matches tissue formation, ensuring sufficient mechanical support while allowing natural bone to regenerate [16,17]. In vitro biodegradation studies are essential to predict the performance of these scaffolds in vivo. Simulated physiological conditions using solutions such as saline, Hank’s balanced salt solution, and phosphate-buffered saline provide valuable insights into how these materials will behave in the body, especially in terms of ion exchange, pH changes, and weight loss [5]. The effect of solution composition on the degradation behavior of PLA-PHB blends can directly impact their suitability as bone substitutes, as changes in pH and ion concentration influence both the scaffold’s mechanical integrity and its interaction with surrounding tissue [18]. In Balogová et al.’s study, a polymer-based material composed of an 85:15 mixture of PLA and PHB was developed with 10% hydroxyapatite and 10% tricalcium phosphate incorporated to enhance its properties. These samples were subjected to in vitro degradation by immersing them in three different solutions: physiological solution, phosphate-buffered saline, and Hanks’ solution. The results indicated that porous samples absorbed 8.33% of the solution, while solid samples absorbed 7.07%, showing a difference of 1.26%. The smallest pH variation from the reference value of 7.4 occurred in the PBS solution, indicating better pH stability [19]. Rahmatabadi et al. investigations into shape memory polymer (SMP) blending, 4D printing, and cold programming (CP) provided additional insights into enhancing the shape memory effect (SME) and functionality of porous structures for medical applications. Similar to our study’s focus on blending PLA with OLA to control degradation, this research demonstrates that blending PLA with thermoplastic polyurethane (TPU) and utilizing CP can improve the mechanical properties and shape recovery of porous structures. The use of SEM to analyze morphology and the results showing high shape recovery ratios and a glass transition temperature shift further emphasize the benefits of polymer blending. These findings align with our approach to optimizing PLA-based materials for biomedical scaffolds, highlighting the importance of material blending in achieving desirable mechanical and functional properties [20,21].

## 2. Materials and Methods

**Figure 1:**
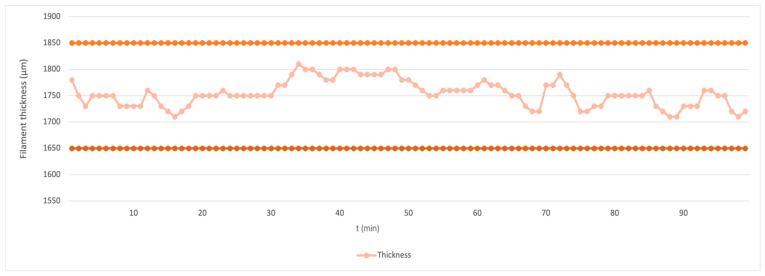
Graph of filament diameter with 0% OLA amount over time (t).

In this study, three different types of samples from commercial products PLA (Mn = 110.000 Da; D-isomer; Purasorb^®^, Amsterdam, The Netherlands), PHB (Mw = 426.000 Da; Biomer P300, Frankfurt, Germany), and OLA (Mn = 957 g mol^−1^; L-isomer; Corbion, Amsterdam, The Netherlands) were produced using the FDM method from granules according to the studies of Arrieta et al. and Burgos et al. [11] [22]. Two blends contained plasticizer OLA at concentrations of 5 wt% and 10 wt%, while the third blend was unplasticized. The material used for producing biomedical filaments was supplied in the form of granules and vacuum sealed. The material was additionally dried using a moisture analyzer Radwag 50/1 (RADWAG, Radom, Poland), with the drying temperature set to 80 °C for 60 min. By optimizing the extrusion conditions—specifically, the temperature, screw rotation speed, and fan performance—the filament was produced with the required quality, maintaining a diameter within a tolerance of 0.1 mm (Figure 1). The filament production process was carried out using a Filament Maker Composer 450 (3devo, Utrecht, The Netherlands) with a temperature range of 165–180 °C. The screw speed was set to 2.5 RPM with 75% fan power for the samples containing 0% and 10% OLA, while for the 5% OLA sample, the screw speed was adjusted to 3.5 RPM with 80% fan power. These settings were determined based on our previous experience and real-time observations of the extrusion process. The process took several hours, starting with preparing and cleaning the equipment using transit materials. Winding the filament onto the spool began only once a stable flow and the correct diameter were reached. Approximately 300 g of material was used during the initial pre-winding phase, which was later recycled and reused in the next steps. The production took place in an air-conditioned room at 18 °C, under sterile laboratory conditions.

**Figure 2:**
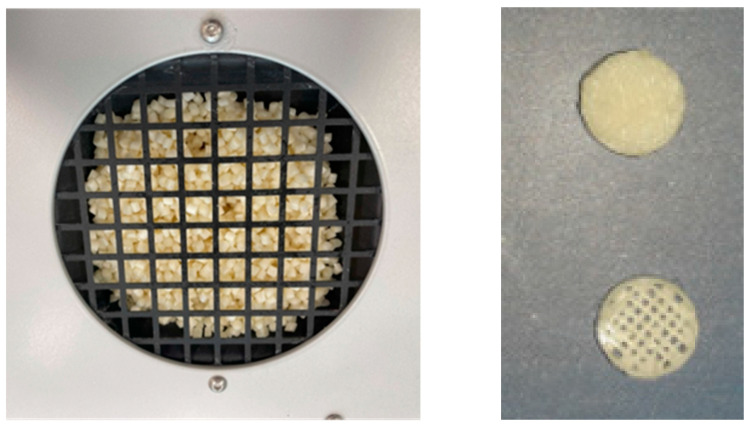
(**Left**)—mixture of PLA/PHB and OLA in pellets, (**Right**)—printed samples solid/porous.

The specimens were prepared in two structural forms: full and porous (Figure 2). To investigate in vitro biodegradation, the samples were immersed in three solutions: saline (A), Hank’s balanced salt solution (B), and phosphate-buffered saline (C). The biodegradation process was monitored over four months by measuring pH changes and weight loss accompanied by atomic absorption spectroscopy (AAS), scanning electron microscopy (SEM), and energy-dispersive spectrometry (EDS).

### 2.1. Production of Samples by 3D Printing

Sample production was conducted at the Department of Biomedical Engineering and Measurement, Faculty of Mechanical Engineering, Technical University of Košice. Six sample types were fabricated using 3D printing technology, consisting of three PLA/PHB (70%/30%) blends and two printing structures (solid and porous). The three material formulations included varying concentrations (OLA as a plasticizer: Material 1 (MAT 1) without OLA, Material 2 (MAT 2) with 5% OLA, and Material 3 (MAT 3) with 10% OLA. The specifications for these materials are summarized in Table 1.

The investigated samples were printed using a TRILAB DeltiQ 2 3D printer (TRILAB Group, Hradec Kralove, Czech Republic, software version 0.1.6), operating on the FDM principle with filament input. The granulate used for filament production was dried with an Airid Polymer dryer (3devo B.V., Utrecht, The Netherlands) at 80 °C for 60 min to prevent moisture-related defects. Sample design and modeling were performed using SolidWorks software (Dassault Systèmes SE, Vélizy-Villacoublay, France). Cylindrical samples, 6 mm in diameter and 2 mm in height, were modeled with a fiber distance of 0.6 mm for solid and 1.2 mm for porous structures. Over 800 samples were printed and print settings (Table 2) were processed using Simplify software (Simplify Technology Group Ltd., Alcester, UK).

### 2.2. Degradation Analysis—Simulation of Physiological Processes

The experimental design employed natural in vitro biodegradation to simulate the physiological processes occurring after implantation of these materials in the human body. An alternative approach, accelerated in vitro biodegradation, was also considered, wherein the materials are subjected to more stringent conditions through increased agitation via mechanical stirring, elevated ambient temperatures, and variations in pH levels during experimentation.

Both methodologies conform to the guidelines outlined in the European Standard ISO 10993-13 (Biological evaluation of medical devices—Part 13: Identification and quantification of degradation products from polymeric medical devices, *International Organization for Standardization (ISO)*, Geneva, Switzerland, 2010) which stipulates parameters such as pH, ambient temperature, and duration of exposure. The primary objective of these experiments was to monitor the absorption characteristics of the solutions and to measure changes in the mass of the tested samples.

In the natural biodegradation experiments, three distinct solutions were utilized for immersion of the samples, labeled as Solutions A, B, and C.

Solution A was a physiological saline solution, which is a 0.9% aqueous sodium chloride solution (9 g NaCl per liter), exhibiting osmolality comparable to that of blood plasma. This solution serves as a vehicle for certain medications and is utilized for various physiological applications. The solution was purchased from EnviroLab s.r.o., Bratislava, Slovakia.

Solution B was Hank’s balanced salt solution (HBSS), an artificial medium commonly employed in laboratory biodegradation testing, providing stable physiological pH and supporting cellular maintenance in a CO_2_-free environment. Its composition is shown in Table 3. The solution was from Merck Life Science spol.s r.o., Bratislava, Slovakia.

Solution C was phosphate-buffered saline, which maintains a constant pH and isotonic conditions. PBS is a mixture of ultra-pure phosphate buffers and pH-adjusted saline solutions, which, after dilution to the working concentration, contains NaCl, KCl, Na_2_HPO_4_, and KH_2_PO_4_ (Table 4). This solution is commonly used in biological research. The buffer helps maintain constant pH levels. Osmolality and concentrations of ions in solutions correspond to the concentrations of the human body.

As a result, 3 sets of flasks were prepared: 3 flasks with solution A, 3 flasks with solution B, and 3 flasks with solution C. The amount of solution in each flask was the same, 42 mL, and the pH value of the individual solutions was 7.4. Banks with solutions and samples were placed on the platform of the Orbital Shaker PSU-10i device (BioSan, Riga, Latvia), which simulated the flow of liquids with its movement. The stirring speed was set at 160 rpm. The Orbital Shaker was placed in an Eeco CelCulture CO_2_ Incubator (Esco Micro Pte. Ltd., Singapur, Singapore), where a constant temperature of 37 °C was maintained throughout the degradation experiment. The experiment lasted 122 days, i.e., 4 months. The samples were weighed before the start of the experiment and during the experiment at regular 30-day intervals on the moisture analyzer (Radwag PMR 50/NH, 50 g, METTLER TOLEDO Corp., Hamilton, New Zealand). Excess liquid was removed from the samples with filter paper. In addition to weighing the samples, the pH of each solution was measured at the same intervals on a Mettler Toledo instrument (METTLER TOLEDO Corp., Hamilton, New Zealand). The pH value was adjusted to 7.4 in the beginning and after each measurement. Solid and porous samples from the same material were placed together in one flask and, after the specified interval, were subsequently separated for the purpose of tests. The measured pH values of the solutions were measured at 30-day intervals.

### 2.3. Chemical Analysis of Samples

Chemical analysis of the samples was performed using the three methods of atomic absorption spectroscopy (AAS), scanning electron microscopy (SEM), and energy-dispersive spectrometry (EDS). The analysis was carried out at the Institute of Geotechnics of the Slovak Academy of Sciences, v. v. i. Košice (ÚGt SAS), Slovakia.

#### 2.3.1. Atomic Absorption Spectroscopy (AAS)

The atomic absorption spectroscopy method was applied to determine the concentration of chemical elements in the analyzed solutions. It is an optical method of analytical chemistry that works on the principle of measuring the absorption of electromagnetic radiation with a wavelength of 190–850 nm by free atoms of the analyzed sample in the gas phase. Flame AAS was measured on a VARIAN AA240 FS (Agilent Technologies, Santa Clara, CA, USA) instrument, where the analyte samples are atomized in a flame of burnt gas (a mixture of acetylene and air). The whole system is set up so that the combustion of the sample and the emission of atoms occur directly in the path of the spectrometer beam. The sample is transported to the flame using a pneumatic capillary nebulizer, which uses the flow of combustion gas (acetylene) and oxidizing agent (air) to cause the sample solution to be sucked in.

#### 2.3.2. Scanning Electron Microscopy (SEM)

The external morphology of the particles, size, shape and their distribution as well as the shape of the pore spaces of the studied samples were investigated using a scanning electron microscope TESCAN MIRA 3 FE SEM (TESCAN, Brno, Czech Republic) by setting an accelerating voltage of 15 to 20 kV at different magnifications. The samples were attached to carbon tape and placed on the instrument holder. SEM analysis did not require covering the samples with a conductive substance.

#### 2.3.3. Energy-Dispersive Spectrometry (EDS)

Energy-dispersive spectrometry (EDS) analysis on selected samples was performed by area distribution of elements—mapping using an additional Oxford Instruments EDS microanalyzer to a TESCAN MIRA 3 FE SEM scanning electron microscope. EDS is an analytical technique used to determine the elemental composition of materials by detecting characteristic X-rays emitted from a sample when it is bombarded with high-energy electrons. Typically paired with scanning or transmission electron microscopy (SEM/TEM), EDS provides both qualitative and quantitative analysis of elements in a sample.

## 3. Results

### 3.1. Degradation Analysis

The biodegradation behavior of three PLA-PHB blends with varying OLA plasticizer concentrations (0%, 5%, 10%) was monitored over a 120-day period in three distinct solutions: saline (A), Hank’s solution (B), and phosphate-buffered saline (C). The pH values of the immersion media were measured at 30-day intervals, while the weight loss of each sample was periodically recorded to assess material degradation. Initial observations indicated that the pH values varied significantly across the three solutions, with distinct trends emerging for each material and solution combination.

#### 3.1.1. Measurement of the Effect of Degradation on Changes in pH Values

The obtained data present pH measurements for three types of materials over four time intervals (30, 60, 90, and 120 days) across three different solutions: saline solution (A), Hank’s solution (B), and phosphate-buffered saline (C) (Table 5).

For the obtained pH values in the saline solution, the pH values for MAT 1, MAT 2, and MAT 3 generally decrease over time, indicating a trend toward more acidic conditions. For Hank’s solution, the pH values for all materials also decrease significantly, particularly for MAT 1, which drops from 3.85 at 30 days to 1.85 at 120 days. This suggests the development of a strong acidic environment. In contrast, the pH values for all materials in phosphate-buffered saline remain relatively stable and are significantly higher than those in saline and Hank’s solutions, showing only slight fluctuations around neutral pH levels.

In the saline solution (A), the pH exhibited a gradual decline across all three materials, indicative of material degradation and subsequent acidification of the surrounding medium. MAT 1 showed a steady drop from an initial pH of 2.36 at 30 days to a low of 2.03 at 90 days, followed by a slight recovery to 2.13 at 120 days. A similar trend was observed in MAT 2, which contained 5 wt% OLA, where the pH decreased from 2.49 at 30 days to 2.04 at 90 days, and then increased to 2.08 by day 120. Notably, MAT 3, with 10 wt% OLA, demonstrated greater pH stability, with values remaining relatively steady between 2.38 and 2.44 from 30 to 90 days, before a slight decrease to 2.34 at 120 days. This suggests that the presence of a higher concentration of plasticizer may have a buffering effect, moderating the acidification caused by material degradation.

In Hank’s balanced salt solution (B), a more pronounced pH reduction was observed, indicating a more aggressive degradation process. MAT 1 exhibited a sharp drop in pH from 3.85 at 30 days to 1.85 by the end of the experiment at 120 days. This substantial acidification suggests rapid degradation of the material, likely due to the release of acidic byproducts into the solution. MAT 2 followed a similar trend, with pH values decreasing from 3.97 at 30 days to 2.29 at 120 days, although the presence of 5 wt% OLA appeared to moderate the rate of acidification. MAT 3, with 10 wt% OLA, demonstrated the slowest pH decline, with values decreasing from 4.36 at 30 days to 2.00 at 120 days, further supporting the hypothesis that the plasticizer slows the degradation process, thereby reducing the extent of pH changes.

In contrast, the phosphate-buffered saline (C) solution exhibited noticeable pH stability across all materials, reflecting the buffering capacity of the solution. For MAT 1, the pH remained relatively constant, starting at 6.09 at 30 days and increasing slightly to 6.33 at 120 days. MAT 2 displayed similar stability, with pH values fluctuating between 6.27 and 6.48 over the course of the experiment. MAT 3, containing the highest concentration of OLA, also showed almost minimal pH variation, with values ranging from 6.78 at 30 days to 6.59 at 120 days. This consistent pH behavior in PBS indicates that the buffered environment effectively neutralized any acidic byproducts released during degradation, thereby maintaining near-neutral conditions throughout the experiment.

#### 3.1.2. Comparison of the Weights of the Dried Samples with the Weights of the Samples Before Degradation

**Figure 3:**
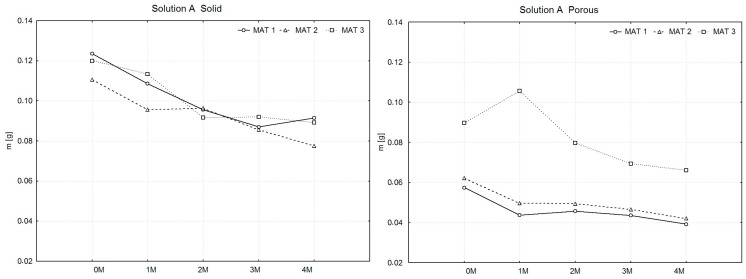
Presentation of the weight changes of dried PLA-PHB 70:30 samples, both solid and porous, following degradation in solution A over a period of four months.

**Figure 4:**
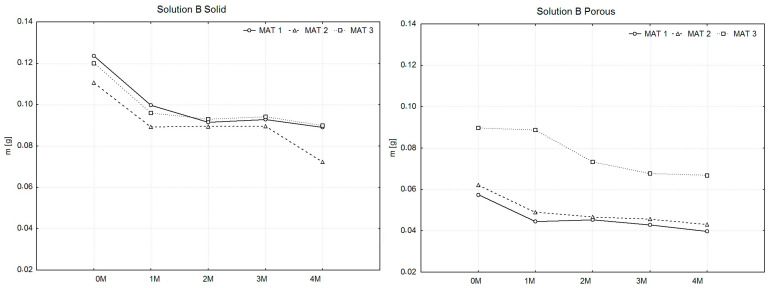
Presentation of the weight changes of dried PLA-PHB 70:30 samples, both solid and porous, following degradation in solution B over a period of four months.

**Figure 5:**
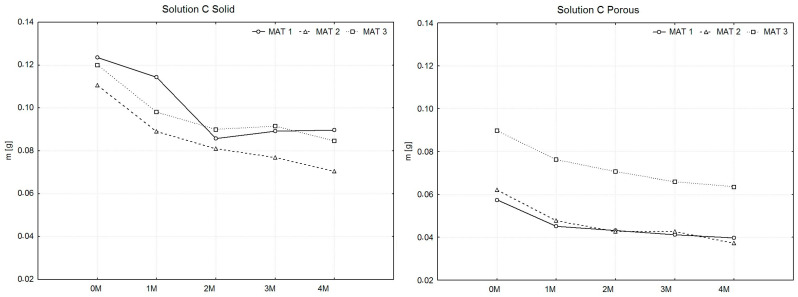
Presentation of the weight changes of dried PLA-PHB 70:30 samples, both solid and porous, following degradation in solution C over a period of four months.

To facilitate the aforementioned comparison, weight measurements (Weighing No. 0) of all produced samples were conducted prior to their immersion in the solutions. The samples were divided into subsets, with the number of samples within each material type ranging from 13 to 15. For these subsets, average weight values were calculated, from which the arithmetic mean X^−^ of the sample averages was subsequently determined for each material type. Similarly, the measured weight values of the samples after drying were processed. These samples were grouped into a single subset for each material type. From the organized values, arithmetic means of the weights were calculated for each material type. To compare the weights of the samples after drying, graphs were created, as illustrated in Figure 3, Figure 4 and Figure 5.

In Figure 3, the solid samples (left graph) display a gradual decrease in mass across all materials. MAT 1 exhibits the most consistent weight loss throughout the four-month period. MAT 2 follows a similar trend but with a slightly lower rate of mass reduction compared to MAT 1. Interestingly, MAT 3 shows an initial decrease in mass over the first two months, followed by a stabilization phase, indicating a slower degradation process as the plasticizer content increases. For the porous samples (right graph), a different pattern is observed. MAT 1 and MAT 2 maintain relatively stable masses over time, with only slight variations. However, MAT 3 demonstrates an initial mass increase after one month, followed by a gradual decline in weight over the subsequent months. This indicates a possible initial absorption of the solution by the porous structure, followed by degradation in later stages.

In Figure 4, the solid samples (left graph) show a consistent pattern of initial mass loss, followed by a phase of stabilization across all materials. MAT 1 shows a steady decrease in weight, while MAT 2 experiences a more rapid mass loss between months one and two before stabilizing. MAT 3 demonstrates a significant decrease in mass during the first month, after which the mass stabilizes, showing a slower degradation process in the later months. For porous samples (right graph), MAT 1 and MAT 2 exhibit a more pronounced initial mass loss over the first month, followed by stabilization in the later months. MAT 3 displays an irregular pattern, with a slight initial increase in mass after one month, possibly due to solution absorption, followed by a gradual decrease.

In Figure 5, the solid samples (left graph) demonstrate a distinct pattern of mass loss across all materials. MAT 1 and MAT 2 show similar degradation profiles, with MAT 1 exhibiting a consistent decrease over time, while MAT 2 experiences a more rapid mass loss in the initial two months before stabilizing. MAT 3 demonstrates a distinct behavior, with a sharp decline in mass during the first month, followed by a slower reduction and eventual stabilization, indicating that higher plasticizer content slows down the degradation process after the initial phase. For porous samples (right graph), a general trend of gradual mass reduction is evident. MAT 1 and MAT 2 follow a nearly identical path, showing steady weight loss across the four months. MAT 3, however, behaves differently, with a more gradual decline in mass, particularly in the later months, suggesting a reduced degradation rate due to the higher plasticizer content.

#### 3.1.3. Comparison of Wet Sample Weights with Sample Weights Before Degradation

**Figure 6:**
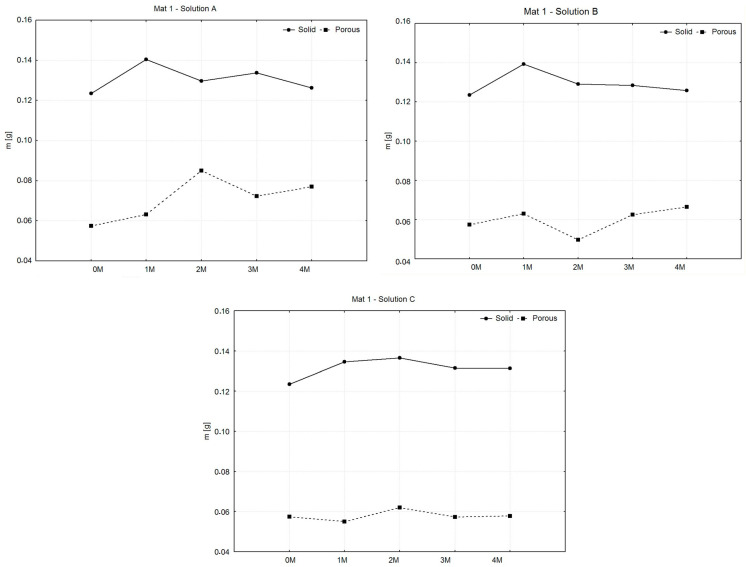
Graphic representation of average weights of MAT 1.

**Figure 7:**
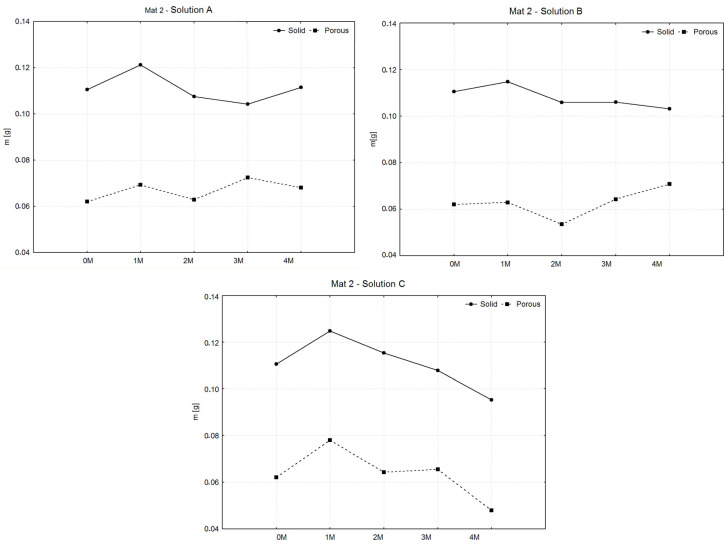
Graphic representation of average weights and variances of MAT 2.

**Figure 8:**
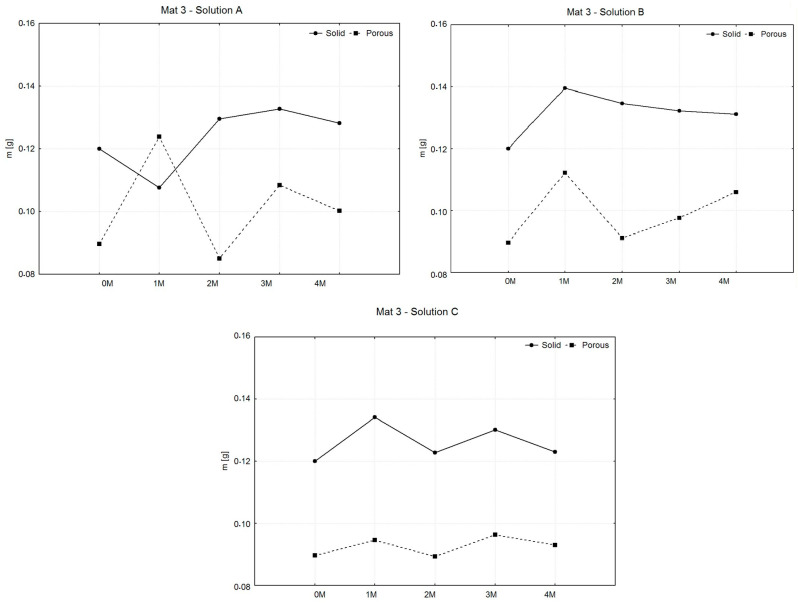
Graphic representation of average weights and variances of MAT 3.

The following comparison of the weights of the wet samples during the entire degradation period served to investigate the ability to increase the weight of the material by absorbing liquid, the amount of which generally affects the intensity of the degradation of the material. The graphs shown in Figure 6, Figure 7 and Figure 8 compare the averages of the average weights of the examined samples in the intervals j = 0, …, 4 (0—before degradation and 1 M–4 M means: during degradation). The statistical measure $x\overset{̿}{j}$ was used because the samples were grouped into several groups (selections) within the same category. The number of sample sets for all types of materials decreased during the experiment due to the separation of samples for related types of tests.

As in Figure 6, in all solutions, solid samples exhibited an initial increase in mass during the first month, followed by minor fluctuations and relative stability for the remainder of the experiment. Porous samples displayed greater variability, with significant mass fluctuations, particularly in saline solution, where the largest changes occurred. Hank’s solution induced more degradation in both sample types, while phosphate-buffered saline caused the least change, indicating a more stable environment. Porous samples consistently showed more weight loss compared to solid samples, likely due to their increased surface area.

Figure 7 shows that in all cases, the solid samples showed an initial increase in mass during the first month, followed by gradual decreases or stabilization. In saline solution, solid samples fluctuated slightly but remained more stable than porous samples, which exhibited small but more variable weight changes. In Hank’s solution, solid samples showed minor fluctuations, while porous samples experienced a more pronounced drop in mass at 2 months, followed by recovery. In phosphate-buffered saline, both solid and porous samples steadily lost weight, with porous samples showing the most significant degradation by the fourth month. Overall, porous samples displayed greater weight loss across all solutions, indicating higher susceptibility to degradation compared to solid samples.

In Figure 8, solid samples show a gradual weight increase, peaking around months 2 or 3, followed by stabilization. In contrast, porous samples exhibit greater variability, with fluctuations in weight across all solutions, especially in solution A. Overall, solid samples maintain higher and more stable weights, while porous samples respond more sensitively to solution conditions, showing greater fluctuations in degradation behavior.

#### 3.1.4. Comparison of Absorption Properties of Materials

The standard way of evaluating the absorption capacity of biodegradable materials is determined using the absorption percentage S_w_.

$$S_{w}=\frac{\left(w_{wet}-w_{dry}\right)}{w_{dry}}\;\cdot \;100\;\left[\%\right]$$

**Figure 9:**
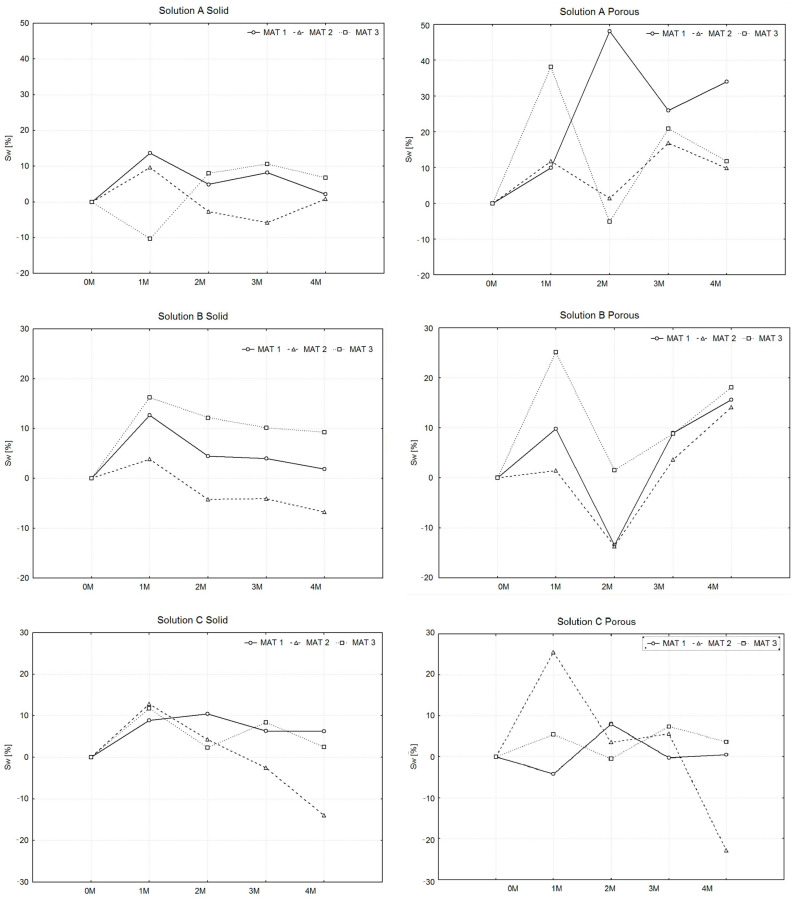
Illustration of the absorption properties of the materials PLA-PHB 70:30 (MAT 1, MAT 2, and MAT 3) in both solid and porous forms across three different solutions (A, B, and C), with absorption measured as swelling percentage over time. The general trend shows that materials with higher plasticizer content (MAT 3 with 10%) demonstrate greater and more consistent liquid absorption, while MAT 1, with no plasticizer, exhibits the lowest absorption and the greatest variability, particularly in its porous form.

The relationship gives the ratio between the weight of the sample w_dry_ before the absorption test and the weight of the sample after it is removed from the solution w_wet_. From the corresponding values, the absorption percentage values were calculated, which are visualized in Figure 9 in the form of graphs comparing individual materials and solutions. For solid samples across all solutions, MAT 1 consistently shows the least absorption, with negative values at some time points, indicating a potential loss of absorbed liquid. In contrast, MAT 3 tends to absorb the most liquid, with absorption stabilizing over time. In all three solutions, the solid samples of MAT 2 and MAT 3 show an initial increase in absorption during the first two months, followed by either stabilization or a slight decrease, suggesting that the higher plasticizer content enhances both the materials’ absorption capacity and their stability over time. The porous samples, on the other hand, show a much higher variability in absorption, particularly MAT 1. For example, in solution A, porous MAT 1 exhibits a sharp increase in absorption, peaking around month 1 with a swelling percentage of 40%, followed by a significant drop and further fluctuations. This pattern suggests that the lack of plasticizer in MAT 1 makes the material more sensitive to environmental changes, leading to less predictable absorption behavior. Conversely, MAT 2 and MAT 3, with added plasticizer, show more stable absorption profiles, though MAT 3, with its higher plasticizer content, tends to absorb more liquid than MAT 2. When comparing the effects of different solutions, solution A induces the most fluctuation in absorption, especially for the porous samples, where the variability is most pronounced in MAT 1. In solution B, the solid samples of all materials behave similarly, with MAT 3 absorbing the most and MAT 1 steadily losing liquid after the first month. Porous samples in solution B again show more variability, but the trend is less extreme than in solution A.

Finally, solution C appears to provide a more stabilizing environment for absorption, particularly for MAT 2 and MAT 3. Solid samples of MAT 2 and MAT 3 maintain a relatively high and steady absorption level, while porous MAT 1 shows significant fluctuation, with a sharp drop after an initially high absorption.

The measured values interpreted in Table 6 provide important statistical measures (for example, the median) that allow better identification of the average absorption capacity with porous structure, compared to solid structure samples. In order to verify the statistical significance of these differences, one of the statistical hypothesis testing methods was used—the non-parametric Mann–Whitney U test. The hypothesis assumes that there is no relevant difference between the measured values of solid and porous samples. The result of the test is the *p*-value, which is compared with the significance level α = 0.05. The results are recorded in Table 3. Based on the testing results, the null hypothesis (*p* > α) could not be rejected. In a similar way, it is possible to verify the influence of individual solutions and materials on the absorption capacity of the samples. For this, the non-parametric Kruskal–Wallis H test was used (Table 7), which tests the null hypothesis, according to which there are no differences between several sample sets, i.e., sets coming from the same base file. Table 5 shows the *p*-values from the comparison of differences between solutions and materials.

The Kruskal–Wallis test results show that the porous form of MAT 1 (0% plasticizer) has a statistically significant difference (*p* = 0.02639) in behavior across the three solutions, indicating that it is highly sensitive to solution type. In contrast, the porous and solid forms of MAT 2 (5% plasticizer) and MAT 3 (10% plasticizer) show no significant differences in behavior across solutions, suggesting that the plasticizer stabilizes these materials, making them less sensitive to environmental changes. Solid samples across all materials show no significant differences, indicating that solid structures are generally more resistant to the effects of solution type compared to porous structures. Overall, the plasticizer enhances stability, particularly in porous samples, while the porous form of MAT 1 is the most affected by solution variation. The lower absorption levels in solid samples are primarily due to their reduced surface area, fewer pathways for ion penetration, and greater resistance to hydrolytic degradation. The compact structure limits interaction with the surrounding solution, and the presence of plasticizer in certain samples further enhances this effect, resulting in slower absorption and greater material stability compared to porous forms. These factors together contribute to the lower overall absorption observed in the solid samples during the study.

### 3.2. Chemical Analysis of Samples

#### 3.2.1. Atomic Absorption Spectroscopy (AAS)

The presence of sodium was detected in all investigated samples, including control. When comparing the samples, it can be seen that all the samples that underwent the biodegradation process had a clearly higher sodium presence compared to the control sample that was not subjected to the biodegradation process (Table 8). This finding is consistent with all solutions containing sodium. Sample MAT 1 in solution C had the highest absorbency for sodium (4400 ppm).

Potassium was present only in the samples that were immersed in solution B and solution C. This finding corresponds to the fact that both of these solutions contain potassium. In samples MAT 1 (C), MAT 2 (C), and MAT 3 (C), the measured presence of potassium was higher than in samples MAT 1 (B), MAT 2 (B), and MAT 3 (B). Sample MAT 1 (C) had the highest absorbability for potassium (730.5 ppm).

The presence of magnesium was confirmed only in samples that were immersed in solution B and solution C. The highest absorbability for magnesium was sample MAT 1 (C) (602.2 ppm).

Calcium was also present only in samples that were immersed in solution B and solution C. Samples made from MAT 2, specifically samples MAT 2 (B) and MAT 2 (C), had a significantly lower measured presence of the chemical element Ca compared to samples made from the other two materials. Samples in solution C had the highest measured amount of chemical elements (Na, K, Mg, and Ca) present.

#### 3.2.2. Scanning Electron Microscopy (SEM)

**Figure 10:**
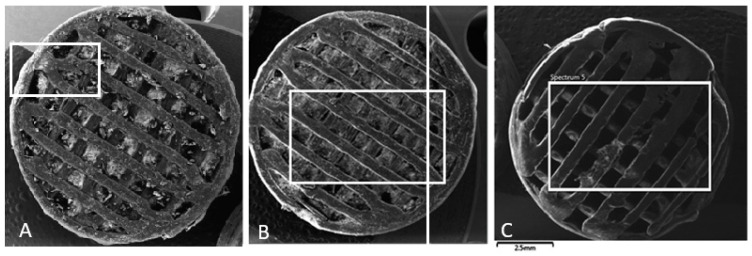
SEM images of MAT 1 in solutions (**A**)—physiological saline solution (**B**)—Hank’s balanced salt solution (HBSS); (**C**)—phosphate-buffered saline (PBS). White rectangle represent a section, from which energy-dispersive spectrometry was performed.

**Figure 11:**
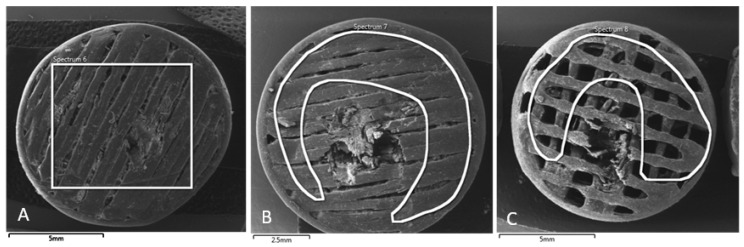
SEM images of MAT 2 in solutions (**A**)—physiological saline solution (**B**)—Hank’s balanced salt solution (HBSS); (**C**)—phosphate-buffered saline (PBS). White rectangle represents a section, from which energy-dispersive spectrometry was performed.

**Figure 12:**
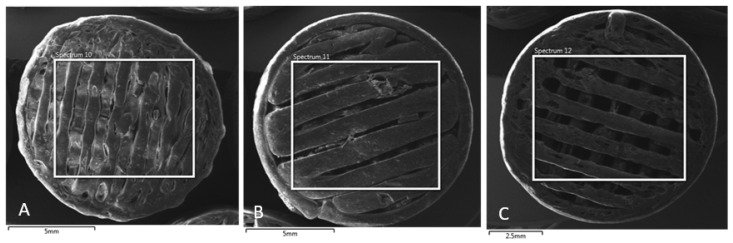
SEM images of MAT 3 in solutions (**A**)—physiological saline solution (**B**)—Hank’s balanced salt solution (HBSS); (**C**)—phosphate-buffered saline (PBS). White rectangle represents a section, from which energy-dispersive spectrometry was performed.

The SEM images presented in Figure 10 (MAT 1), Figure 11 (MAT 2), and Figure 12 (MAT 3) were primarily captured to perform energy-dispersive spectrometry (EDS) analysis on the selected regions, marked by white rectangles. The SEM images provide a visual overview of the surface morphology changes that occurred after immersion in the respective solutions, while the white rectangles highlight the specific areas where EDS was conducted to further investigate the elemental composition and chemical changes in these regions.

#### 3.2.3. Energy-Dispersive Spectrometry (EDS)

**Figure 13:**
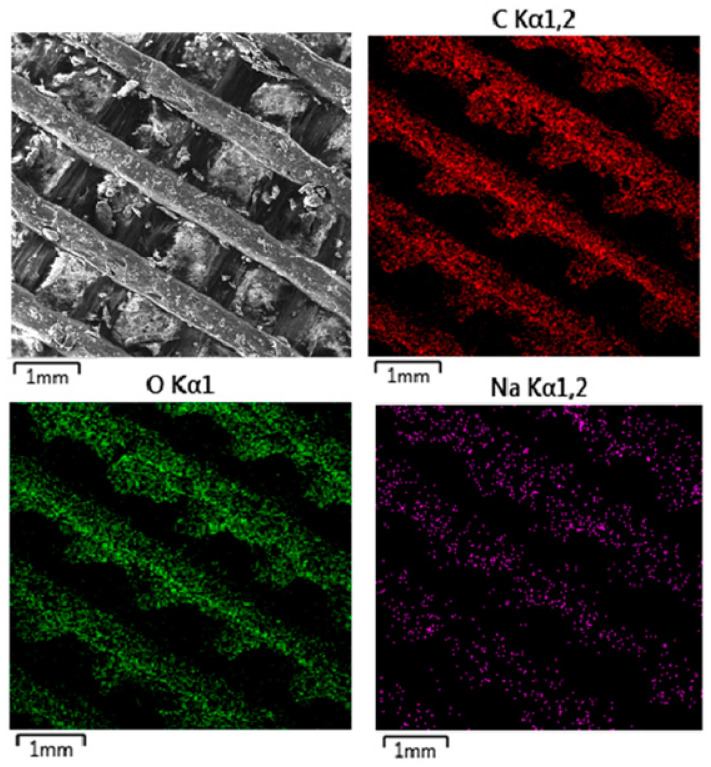
EDS mapping of MAT 1 in solution A. The provided EDS mapping illustrates the elemental distribution of MAT 1 after immersion in physiological saline solution (solution A).

Figure 13 presents EDS mapping of MAT 1 after immersion in physiological saline solution (solution A), highlighting key elemental distributions and interactions with the material. The carbon and oxygen maps show uniform distribution across the surface, indicating that the polymer matrix has retained its structural and chemical integrity after immersion. This suggests that the material did not undergo significant degradation or alteration, maintaining its core composition. The sodium map reveals that sodium ions from the saline solution have adhered to the material’s surface in a non-uniform manner, likely through ionic interactions. However, the concentration of sodium appears localized to the surface, with no evidence of deep penetration or significant impact on the material’s internal structure. The SEM image further highlights some surface roughness and debris, which may be attributed to the minor physical erosion or adsorption of salts from the solution.

**Figure 14:**
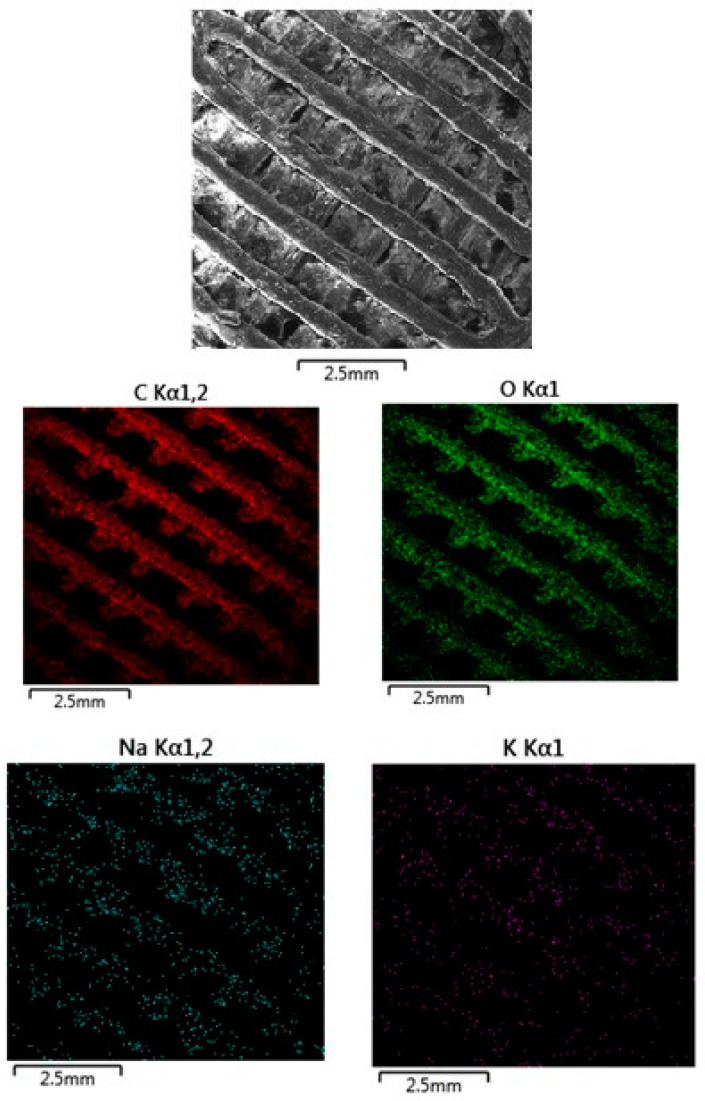
EDS mapping of MAT 1 in solution B. The SEM image shows noticeable surface roughness and debris compared to the material immersed in physiological saline solution, suggesting increased interaction between the material and the HBSS environment.

The EDS map (Figure 14) for carbon, represented in red, indicates uniform distribution across the material. This is expected since carbon is the primary component of the PLA-PHB polymer matrix. The consistent carbon distribution suggests that the polymer structure remains chemically intact after immersion in HBSS, with no significant degradation or loss of carbon from the matrix. The absence of OLA means the carbon content strictly reflects the PLA-PHB material, and the uniformity further supports the conclusion that the polymer matrix has remained stable. Oxygen is present in the polymer matrix due to the oxygen-containing functional groups in PLA and PHB. The uniform oxygen distribution suggests that the ester linkages and oxygen-containing groups have not been disrupted, and the polymer remains chemically stable. There is no sign of oxidation or significant hydrolytic degradation, which corresponds to the relatively smooth appearance of the material in the SEM image. The sodium map, shown in cyan, displays a more concentrated distribution on the surface of the material. Sodium ions, coming from the HBSS solution, have adhered to the surface of the material, but their interaction appears to be limited primarily to the surface level. The distribution suggests a moderate ionic exchange between the sodium ions in the HBSS solution and the polymer surface, but without significant penetration into the deeper layers of the material. The absence of a plasticizer, such as OLA, may reduce the material’s hydrophilicity, limiting sodium absorption compared to samples containing plasticizer. The potassium map, shown in purple, reveals a localized but scattered presence of potassium on the material’s surface. Potassium is another component of HBSS, and its distribution suggests limited interaction with the material compared to sodium. The scattered nature of potassium indicates that it may have less affinity for the polymer surface compared to sodium, resulting in a more sporadic ionic attachment.

**Figure 15:**
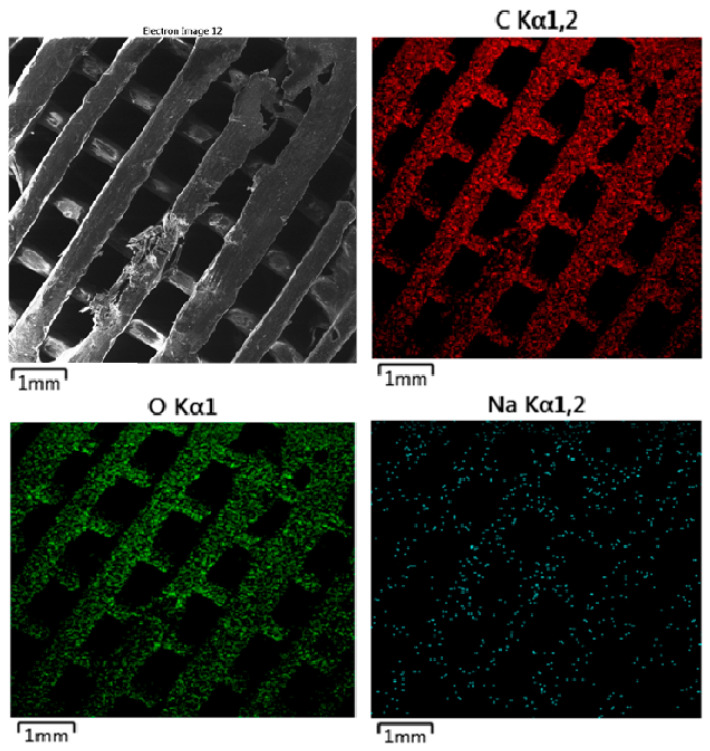
EDS mapping of MAT 1 in solution C. The SEM image reveals more pronounced surface degradation compared to the previous solutions (A and B).

The EDS map (Figure 15) for carbon, shown in red, indicates uniform distribution across the material surface. The consistent carbon distribution suggests that despite the physical surface degradation observed in the SEM image, the polymer matrix remains chemically intact with no significant loss of carbon content. The oxygen map, represented in green, also shows a uniform distribution throughout the material. Oxygen is a key component of the PLA-PHB structure, with ester linkages and oxygen-containing groups in the polymer matrix. The stable distribution of oxygen further indicates that the material’s oxygen-containing functional groups remain intact, despite the surface roughness. This can suggest that the polymer is not undergoing significant hydrolytic degradation in PBS but is instead experiencing surface erosion. The sodium map, shown in cyan, reveals a scattered but evident presence of sodium on the surface of the material. Sodium ions originate from the PBS solution, and their distribution suggests some level of interaction between the ions and the polymer surface. However, sodium does not appear to have deeply penetrated the polymer matrix, indicating that the interaction is primarily at the surface level. Sodium absorption is likely facilitated by the ionic nature of PBS and its slightly acidic pH, contributing to surface degradation and increased roughness.

**Figure 16:**
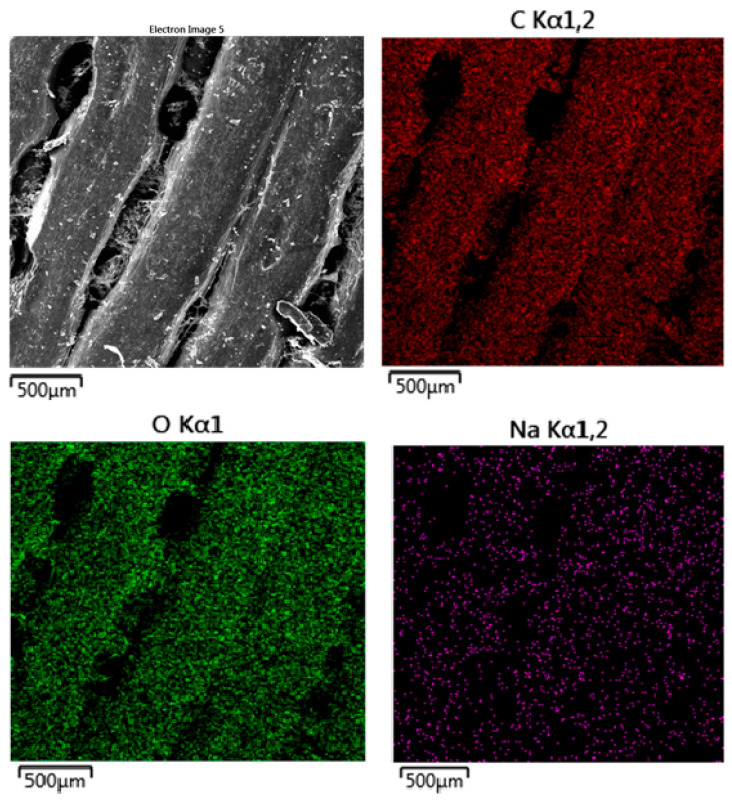
EDS mapping of MAT 2 in solution A. The SEM image reveals a relatively smooth surface with some visible grooves and minor roughness, indicating that the material has maintained its structural integrity after exposure to the physiological saline solution.

The EDS map (Figure 16) for carbon, represented in red, shows a uniform distribution across the surface of the material. Since carbon is a major component of both the PLA-PHB polymer matrix and the OLA plasticizer, the consistent distribution suggests that both the polymer matrix and the plasticizer have remained chemically intact. There is no indication of significant carbon loss or breakdown. The oxygen map, displayed in green, also shows a uniform distribution throughout the material. Oxygen is part of the PLA-PHB structure as well as the OLA plasticizer, both of which contain oxygen in ester groups and other functional groups. The consistent oxygen distribution suggests that these functional groups have not undergone degradation. The retention of oxygen distribution reflects that the material’s oxygen-containing groups, including those from the OLA plasticizer, remain intact, showing no signs of significant hydrolytic degradation. The sodium (Na) map, shown in purple, indicates a sparse and scattered presence of sodium across the surface of the material. Sodium ions originate from the physiological saline solution (sodium chloride). The minimal absorption of sodium suggests limited ionic interaction between the material and the solution. The presence of the 5% OLA plasticizer, which increases the material’s hydrophilicity, may have facilitated some sodium absorption, but this interaction appears to be mostly superficial, with no deep penetration into the polymer matrix.

**Figure 17:**
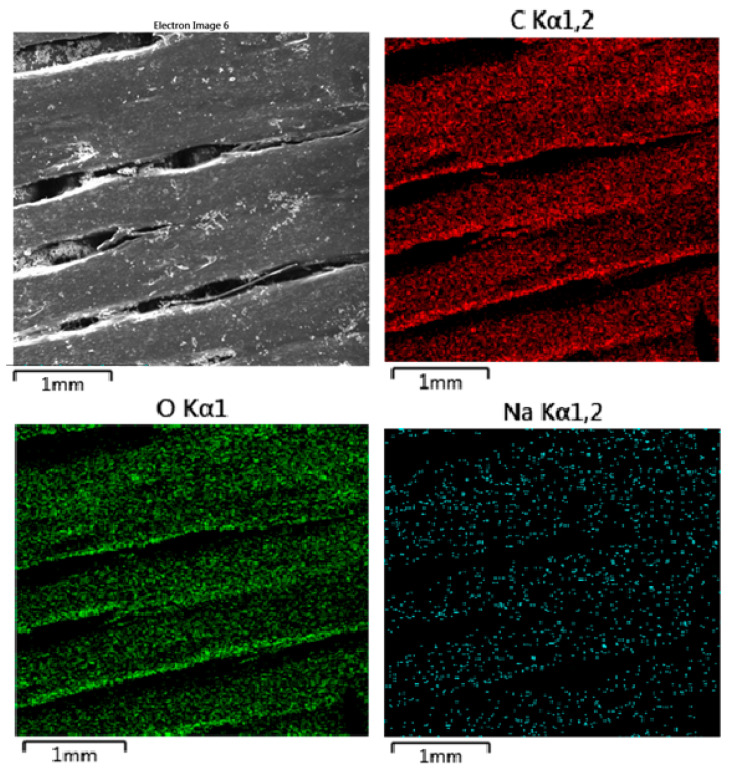
EDS mapping of MAT 2 in solution B. The SEM image shows a relatively smooth surface with visible linear grooves. The material appears mostly intact, though there are minor surface irregularities and signs of cracking along the fiber surfaces.

The EDS map (Figure 17) for carbon, displayed in red, shows a uniform distribution across the surface of the material. The consistent carbon distribution suggests that the polymer matrix remains chemically intact after immersion in HBSS, with no significant loss of carbon content. The oxygen map, represented in green, is also evenly distributed across the material. The uniform oxygen distribution indicates that the material’s ester linkages and oxygen-containing groups remain intact, with no significant breakdown or hydrolytic degradation. The sodium map, shown in cyan, reveals a more concentrated presence of sodium on the surface of the material compared to solution A. Sodium ions come from the HBSS solution, and the increased sodium distribution indicates a stronger ionic interaction with the polymer surface. The presence of sodium may be due to the enhanced absorption of ions facilitated by the hydrophilicity of the OLA plasticizer. However, the interaction appears mostly superficial, with sodium adhering to the surface rather than penetrating deeply into the polymer matrix.

**Figure 18:**
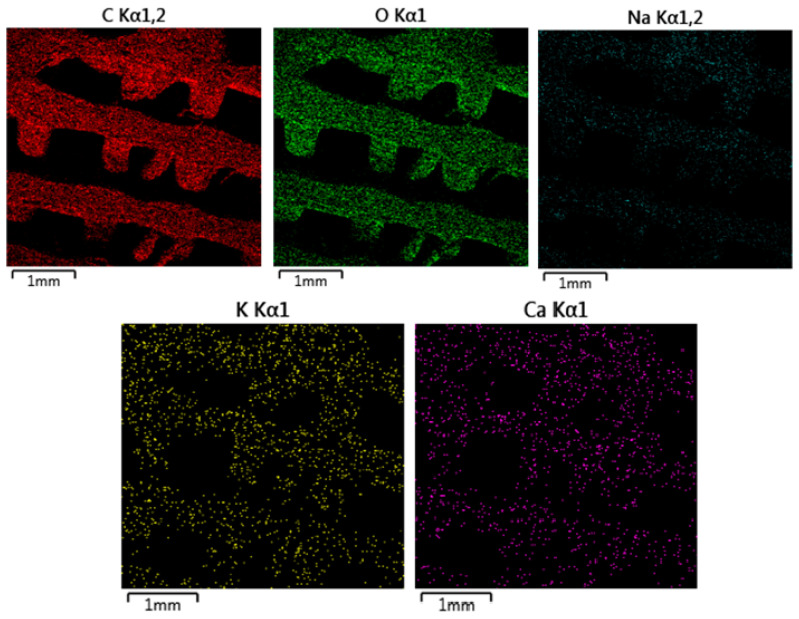
EDS mapping of MAT 2 in solution C. The interaction with ions such as sodium, potassium, and calcium seems to be primarily superficial, with limited ionic absorption and no deep penetration into the polymer.

The EDS map (Figure 18) for carbon, represented in red, shows a uniform distribution across the surface of the material. The consistent carbon distribution suggests that, despite exposure to PBS, the polymer matrix remains chemically intact. This indicates that the material has not experienced significant carbon loss or degradation, and the polymer structure remains stable. The oxygen map, displayed in green, shows a uniform distribution throughout the material. This suggests that the polymer has retained its chemical stability, and there are no signs of major breakdown in the oxygen-containing functional groups. The sodium map, shown in cyan, reveals a sparse distribution of sodium on the material’s surface. Sodium originates from the PBS solution, and its limited presence indicates minimal absorption of sodium ions by the material. The 5% OLA plasticizer, which enhances hydrophilicity, does not seem to have facilitated substantial ionic exchange with sodium in this case, suggesting that the sodium interaction is primarily superficial and does not penetrate deeply into the material. The potassium map, shown in yellow, reveals a scattered distribution of potassium ions across the surface. The distribution of potassium is scattered, indicating localized attachment of ions rather than uniform penetration into the polymer matrix. The calcium (Ca) map, shown in purple, shows a scattered distribution across the material’s surface, similar to potassium. Calcium ions are also present in PBS and appear to have adhered to the surface of the material in a localized manner.

**Figure 19:**
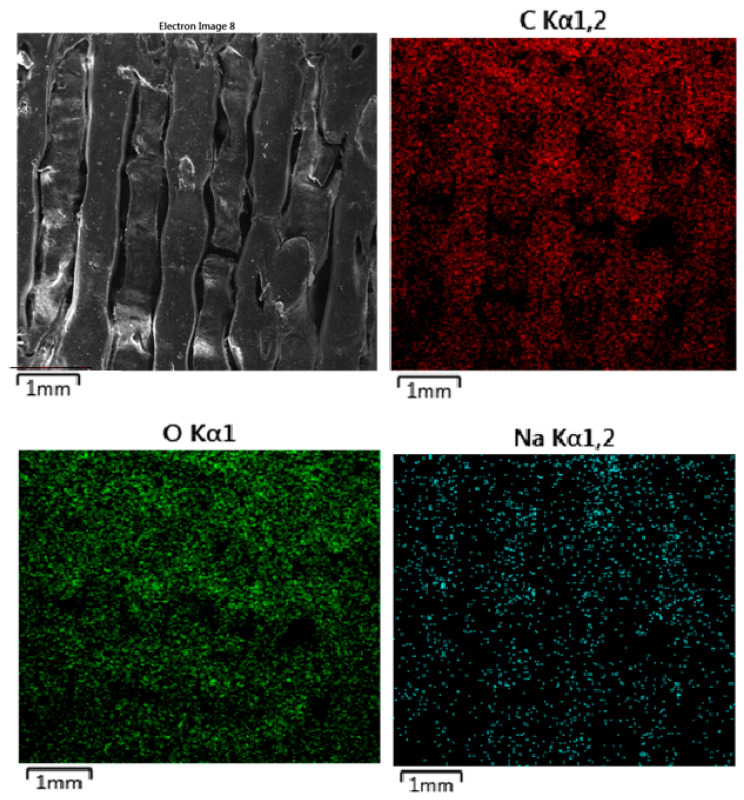
EDS mapping of MAT 3 in solution A. The figure shows the distribution of carbon (C), oxygen (O), and sodium (Na), accompanied by an SEM image illustrating the surface morphology of the material. The SEM image displays a relatively smooth surface with some visible linear grooves and minor roughness, indicating that the material has retained most of its structural integrity after immersion in physiological saline solution.

The EDS map (Figure 19) for carbon, represented in red, shows a uniform distribution across the surface of the material. The material shows no significant carbon loss or breakdown, indicating that it has retained its integrity in this environment. The oxygen map, represented in green, also shows a uniform distribution throughout the material. The absence of any significant variation in the oxygen distribution supports the conclusion that the material has not undergone hydrolytic degradation in the physiological saline solution. The sodium map, shown in cyan, reveals a sparse and scattered presence of sodium ions on the surface of the material. Sodium originates from the physiological saline solution (sodium chloride). Despite the 10% OLA plasticizer, which enhances the material’s hydrophilicity, the interaction with sodium ions appears superficial, and there is no evidence of deep penetration of sodium into the polymer matrix.

**Figure 20:**
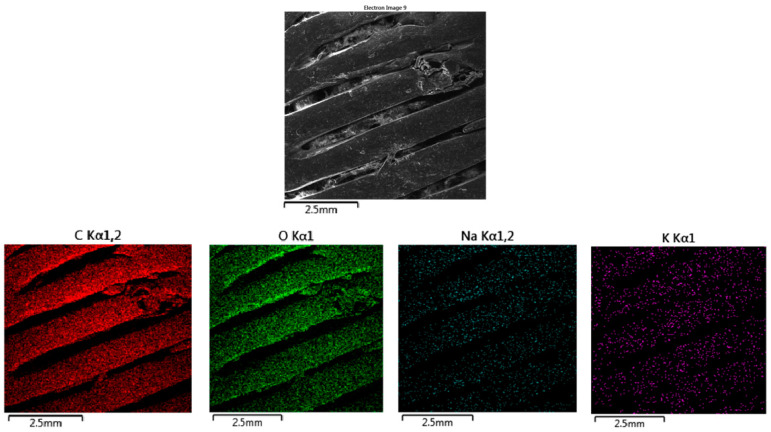
EDS mapping of MAT 3 in solution B. The figure provides the distribution of carbon (C), oxygen (O), sodium (Na), and potassium (K), along with an SEM image showing the surface morphology of the material.

The EDS (Figure 20) map for carbon, displayed in red, shows a uniform distribution across the surface of the material. No significant carbon loss is observed, indicating that the material has retained its structural integrity despite some surface roughness. The oxygen map, represented in green, also shows uniform distribution throughout the material. The consistent oxygen distribution suggests that the oxygen-containing functional groups in the polymer matrix, such as ester bonds, remain intact. The sodium map, shown in cyan, reveals a significant concentration of sodium on the surface of the material. Sodium ions originate from the HBSS solution, and their more concentrated presence suggests a stronger interaction with the polymer surface. The 10% OLA plasticizer, which increases the material’s hydrophilicity, likely enhances the material’s ability to absorb sodium ions. However, the interaction is still largely superficial, with no deep penetration into the polymer matrix. The potassium map, represented in purple, shows a scattered distribution of potassium ions across the material’s surface. Similar to sodium, the interaction with potassium appears to be superficial, with no deep penetration into the polymer matrix.

**Figure 21:**
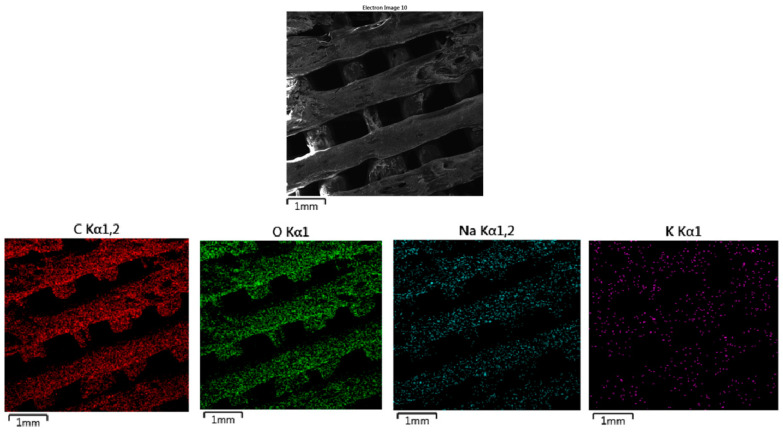
EDS mapping of MAT 3 in solution C. The SEM image shows visible surface grooves and roughness, with areas of apparent surface degradation despite the surface changes, the material’s overall structure remains mostly intact.

The EDS map (Figure 21) for carbon, represented in red, shows a uniform distribution across the surface of the material. The consistent carbon distribution suggests that the polymer matrix remains chemically stable after immersion in PBS. The stable oxygen distribution suggests that the oxygen-containing functional groups (such as ester linkages) remain chemically intact, and there is no sign of substantial hydrolytic degradation. The sodium (Na) map, shown in cyan, reveals a high concentration of sodium on the surface of the material. Sodium ions originate from the PBS solution, and the elevated concentration suggests a significant interaction with the polymer surface. The 10% OLA plasticizer, which increases the material’s hydrophilicity, likely contributes to the enhanced absorption of sodium ions. However, this interaction appears to be superficial, with sodium primarily attaching to the surface rather than penetrating deeply into the polymer matrix. The potassium (K) map, shown in purple, indicates a scattered distribution of potassium ions across the surface of the material.

## 4. Discussion

In this study, we evaluated the absorption properties and degradation behavior of PLA-PHB 70:30 materials (MAT 1, MAT 2, and MAT 3) with varying amounts of plasticizer (0%, 5%, and 10% OLA) in both full and porous forms when immersed in three different solutions: saline (solution A), Hank’s solution (solution B), and phosphate-buffered saline (PBS, solution C). The results reveal critical insights into how plasticizer content, material structure (solid vs. porous), and solution type influence the biodegradation and absorption characteristics of these materials. The analysis of the mass loss over time reveals that the presence of a plasticizer (OLA) plays a vital role in stabilizing the material’s absorption properties. Specifically, MAT 3 (10% plasticizer) demonstrated more stable mass retention across all three solutions compared to MAT 1 (0% plasticizer), which exhibited significant fluctuations, particularly in porous forms. The pH measurements further highlight the differential impact of the plasticizer and solution type on the degradation process. For materials with no plasticizer (MAT 1), the pH values in saline and Hank’s solution dropped significantly over time, indicating a more rapid degradation process and the accumulation of acidic degradation products. This phenomenon is expected, as the degradation of PLA and PHB typically leads to the release of acidic by-products, such as lactic and hydroxybutyric acid, which can lower the pH of the surrounding medium. In contrast, MAT 3 showed more stable pH values over the four-month period, especially in PBS, suggesting that the higher plasticizer content helps to mitigate the release of degradation by-products, likely by reducing the hydrolysis rate and increasing the material’s resistance to degradation. These results are consistent with prior studies that have demonstrated the role of plasticizers in enhancing the flexibility and hydrophilicity of biodegradable polymers, thus improving their interaction with aqueous environments while minimizing rapid degradation. A study by Chaochanchaikul et al. investigated the effects of using ozonized soybean oil (OSBO) as a biobased plasticizer to improve the toughness of PLA. OSBO was synthesized and added to PLA in varying amounts (0–15 wt%). The results showed that increasing OSBO content enhanced the elongation at break and impact strength but reduced tensile strength. The glass transition, crystallization, and melting temperatures of PLA decreased with higher OSBO content, indicating improved flexibility [23]. In Arrieta et al.’s study, acetyl tri-n-butyl citrate (ATBC) was used as a plasticizer to improve the flexibility of PLA-PHB electrospun mats, specifically in the PLA75–PHB25 blend. The addition of ATBC significantly increased the elongation at break, enhancing the ductility required for flexible film applications. This plasticization resulted in improved mechanical properties, making the material more adaptable for flexible uses [11]. In a study by Burgos et al., PLA was melt-blended with varying concentrations of OLA (15 wt% to 25 wt%) to enhance the ductility of PLA and produce a fully biodegradable material for potential use in film manufacturing. The results showed that OLA was an effective plasticizer, significantly reducing the glass transition temperature (Tg) and improving the ductile properties of PLA. Notably, no phase separation was detected, indicating good compatibility between PLA and OLA. Over a 3-month storage period, blends containing 20 wt% and 25 wt% OLA remained stable, with PLA-20 wt% OLA maintaining an amorphous structure and exhibiting optimal thermal, mechanical, and oxygen barrier properties, making it suitable for flexible film production [22].

An additional AAS, SEM, and EDS analysis of our porous samples showed that MAT 1, which contains no OLA plasticizer, experienced the fastest degradation across all tested solutions. In saline, MAT 1 exhibited a drop in pH, indicating accelerated hydrolysis, which was confirmed by SEM and EDS analyses showing surface degradation and sodium absorption. In HBSS, this effect was even more pronounced, with MAT 1 showing a substantial drop in pH to 1.85 by day 120, alongside high levels of calcium, magnesium, and potassium absorption, further driving hydrolytic degradation. Although PBS stabilized the pH due to its buffering capacity, MAT 1 still showed ionic absorption and moderate degradation, indicating that the lack of plasticizer renders the material susceptible to breakdown, particularly in ion-rich environments.

In contrast, MAT 2, containing 5% OLA plasticizer, demonstrated significantly slower degradation in all three solutions. We assume the plasticizer played a key role in moderating ionic absorption and maintaining pH stability. In saline, MAT 2 showed a more gradual pH decline and lower sodium absorption, which translated into slower hydrolytic degradation. In HBSS, the plasticizer again reduced the rate of degradation, with lower calcium and magnesium absorption, resulting in a more controlled pH drop compared to MAT 1. In PBS, MAT 2 exhibited the lowest ionic absorption and maintained a neutral to slightly basic pH, indicating that the plasticizer effectively minimized interaction with ions and delayed the onset of material breakdown.

MAT 3, with 10% OLA plasticizer, proved to be the most stable and resilient material across all solutions, where it showed the greatest mass retention and controlled degradation. The higher plasticizer content significantly reduced water uptake, ionic absorption, and the rate of hydrolytic degradation, making MAT 3 an ideal candidate for applications requiring prolonged stability, such as biomedical scaffolds and tissue engineering. Its ability to maintain pH stability, coupled with its low swelling and minimal mass loss, highlights the critical role of the 10% OLA plasticizer in enhancing the performance of PLA-PHB 70:30 materials.

These results have significant implications for the use of PLA-PHB blends in biomedical and environmental applications, where controlled degradation and predictable absorption properties are crucial. Freier et al.’s study examined the in vitro degradation of solution-cast films of PHB and its blends with modifications, as well as poly(l-lactide) (PLLA), for use in a restorable gastrointestinal patch. The results showed that pure PHB’s molecular weight decreased by half after one year in Sørensen buffer solution. Blending with PHB accelerated degradation, while a hydrophobic plasticizer decelerated it. The plasticizer slowed down the degradation rate by reducing water penetration, whereas water-soluble additives had a slight accelerating effect [24]. Barbeck et al.’s study aimed to analyze the in vitro and in vivo degradation of a bi-layered 3D-printed scaffold combining a PLA layer and a biphasic PLA/bioglass G5 layer for osteochondral defect regeneration. In vitro analysis focused on weight loss, morphological changes, and mechanical variations after immersion in simulated body fluid (SBF). Both scaffold parts maintained structural integrity, but the PLA/G5 scaffold showed more significant morphological changes. The addition of G5 reduced scaffold weight loss and increased the compressive modulus compared to PLA alone. In vivo, the PLA/G5 scaffold induced greater tissue reactions, with higher vascularization and more bone marrow giant cells (BMGCs), essential for bone regeneration, while PLA alone resulted in minimal vascularization, favorable for cartilage regeneration. The results highlight that the solution (SBF) influenced degradation by progressively weakening mechanical properties, but the addition of bioglass G5 mitigated weight loss and promoted better mechanical stability and biological responses necessary for osteochondral regeneration [25].

## 5. Conclusions

The study demonstrates that careful control over the material structure (solid vs. porous) and the addition of plasticizers can tailor the degradation rate to specific application needs. For example, in biomedical scaffolds, porous materials may be preferred for rapid degradation and tissue ingrowth, but plasticizers may be needed to moderate degradation and ensure compatibility with the surrounding environment. The results of this study demonstrate that PLA-PHB blends with varying plasticizer content, particularly with 10% OLA, provide a robust strategy for designing biodegradable scaffolds with controlled degradation suitable for long-term applications in tissue regeneration. Future research could focus on the pore morphology of the scaffolds to further evaluate their impact on cell adhesion, proliferation, and overall biocompatibility. Additionally, in vivo studies would be valuable in assessing the tissue response and scaffold integration in biological systems.

## Figures and Tables

**Table 1:** Characteristics and abbreviations of the materials.

	PLA-PHB 70:30 (MAT 1)	PLA-PHB 70:30 (MAT 2)	PLA-PHB 70:30 (MAT 3)
Structure of the materials	Full	Full	Full
	Porous	Porous	Porous
The amount of added plasticizer—OLA	0%	5%	10%

**Table 2:** Parameters of 3D printing.

Parameter	Value
Nozzle size	0.04 mm
Nozzle temperature	190 °C
Platform temperature	70 °C
Print speed	1500 mm/min
Print time per sample	2 min

**Table 3:** Composition of Hank’s balanced salt solution (HBSS).

Component	Quantity (g)	Concentration
NaCl (Mw: 58.44 g/mol)	8.00	0.1400 M
KCl (Mw: 74.55 g/mol)	0.40	0.0050 M
CaCl_2_ (Mw: 110.98 g/mol)	0.14	0.0010 M
MgSO_4_-7 H_2_O (Mw: 246.47 g/mol)	0.10	0.0004 M
MgCl_2_-6 H_2_O (Mw: 203.303 g/mol)	0.10	0.0005 M
Na_2_HPO_4_-2 H_2_O (Mw: 177.99 g/mol)	0.60	0.0003 M
KH_2_PO_4_ (Mw: 136.086 g/mol)	0.60	0.0004 M
*D*-glucose (Dextrose) (Mw: 180.156 g/mol)	1.00	0.0060 M
NaHCO_3_ (Mw: 84.01 g/mol)	0.35	0.0040 M

**Table 4:** Composition of phosphate-buffered saline (PBS).

Component	Quantity (g)	Concentration
NaCl (Mw: 58.44 g/mol)	8.000	0.1370 M
KCl (Mw: 74.55 g/mol)	0.200	0.0027 M
Na_2_HPO_4_ (Mw: 141.96 g/mol)	1.440	0.0100 M
KH_2_PO_4_ (Mw: 136.086 g/mol)	0.245	0.0018 M

**Table 5:** Time-dependent pH measurements of PLA-PHB 70:30 samples in saline, Hank’s solution, and phosphate-buffered saline. The table presents pH data over four time intervals (30, 60, 90, and 120 days) for three different materials (MAT 1, MAT 2, and MAT 3) immersed in three different solutions.

Sample	Solution	Time of Interval of Measurement of pH Values (Days)			
		30	60	90	120
MAT 1	Saline solution	2.36	2.22	2.03	2.13
	Hank’s solution	3.85	1.91	1.86	1.85
	Phosphate-buffered saline	6.09	6.13	6.27	6.33
MAT 2	Saline solution	2.49	2.09	2.04	2.08
	Hank’s solution	3.97	2.63	2.41	2.29
	Phosphate-buffered saline	6.27	6.18	6.11	6.48
MAT 3	Saline solution	2.38	2.41	2.44	2.34
	Hank’s solution	4.36	2.15	1.94	2.0
	Phosphate-buffered saline	6.78	6.68	6.56	6.59

**Table 6:** Analysis of the absorption results using the non-parametric Mann–Whitney U test.

Solution/Material	MAT 1	MAT 2	MAT 3
*p* (A)	0.05714	0.05714	0.20000
*p* (B)	0.6857	0.6857	0.1143
*p* (C)	0.1143	0.8857	0.6857

**Table 7:** Values from comparison of differences between solutions using Kruskal–Wallis-test.

Sample		Compared Solutions A 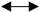 B 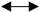 C
MAT 1	Solid	*p* = 0.4374
	Porous	*p* = 0.02639
MAT 2	Solid	*p* = 0.5836
	Porous	*p* = 0.6677
MAT 3	Solid	*p* = 0.1229
	Porous	*p* = 0.3094

**Table 8:** The results of the atomic absorption spectroscopy.

Sample	Na [%]	Na [ppm]	K [ppm]	Mg [ppm]	Ca [%]	Ca [ppm]
Control sample ^1^	0.06	600	BDL ^2^	BDL	BDL	BDL
MAT 1 (A)	0.27	2700	BDL	BDL	BDL	BDL
MAT 1 (B)	0.21	2100	218	555.1	0.58	5800
MAT 1 (C)	0.44	4400	730.5	602.2	0.49	4900
MAT 2 (A)	0.26	2600	BDL	BDL	BDL	BDL
MAT 2 (B)	0.24	2400	218	210	0.24	2400
MAT 2 (C)	0.38	3800	675	275	0.22	2200
MAT 3 (A)	0.21	2100	BDL	BDL	BDL	BDL
MAT 3 (B)	0.26	2600	215.4	482.5	0.49	4900
MAT 3 (C)	0.38	3800	618.3	575.7	0.50	5000

^1^ Non-degraded sample, ^2^ below detecting unit.

## Data Availability

Data is contained within the article. The original contributions presented in the study are included in the article, further inquiries can be directed to the corresponding author/s.
